# Supplementary material for: Sulfated Glycosaminoglycans as Viral Decoy Receptors for Human Adenovirus Type 37
Source: Viruses. 2019 Mar 12;11(3):247. doi: 10.3390/v11030247 (PMC6466042; doi:10.3390/v11030247)
Supplement: Supplementary file 1 [file viruses-11-00247-s001.zip › viruses-459887-final check-supp/Supplementary table S1.pdf]

Table S1. Probes investigated in a focused GAG microarray

Chart Position	Probe[a]	Sequence[b]
1	HA 12-mer	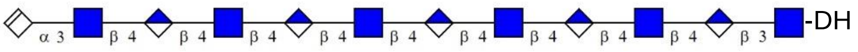
2	CS-A 6-mer	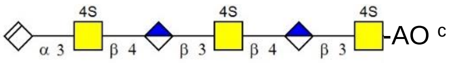
3	CS-A 14-mer	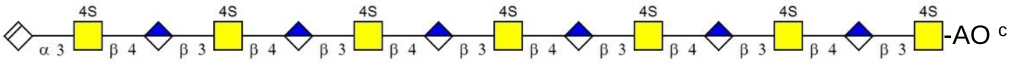
4	CS-B 6-mer	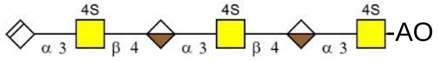
5	CS-B 14-mer	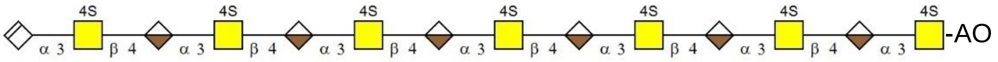
6	CS-C 6-mer	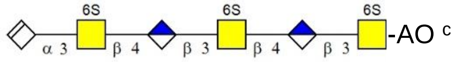
7	CS-C 14-mer	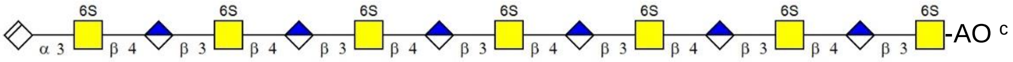
8	Hep 10-mer	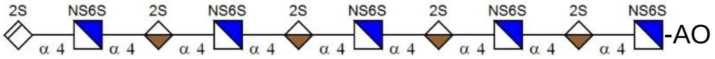
9	Hep 14-mer	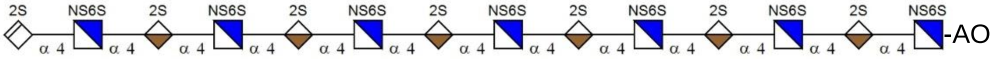
10	HS S6-mer	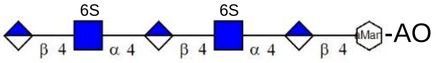 May contain small amounts of GlcNS(6S) and IdoA(2S); some GlcNAc may be non-sulfated
11	HS S8-mer	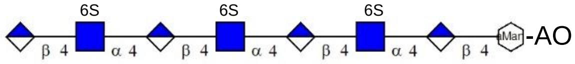 May contain small amounts of GlcNS(6S) and IdoA(2S); some GlcNAc may be non-sulfated
12	KS 6-mer (K'ase I)	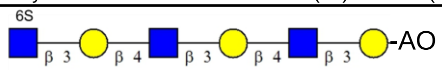 Variously 6S on Gal and GlcNAc
13	KS 14-mer (K'ase I)	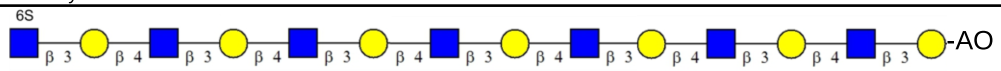 Variously 6S on Gal and GlcNAc
14	KS 6-mer (K'ase II)	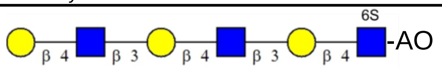 Variously 6S on Gal and GlcNAc
15	KS 14-mer (K'ase II)	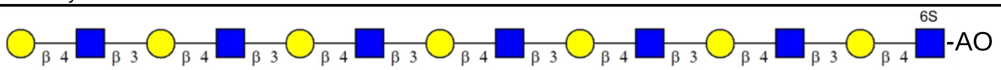 Variously 6S on Gal and GlcNAc

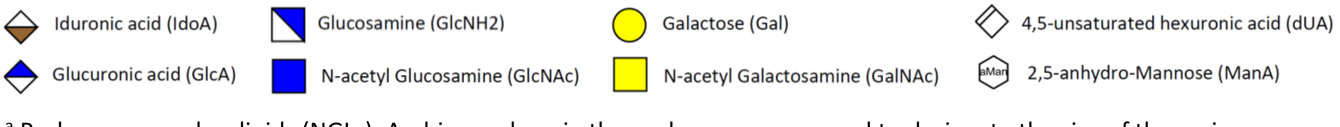

[a] Probes are neoglycolipids (NGLs). Arabic numbers in the probe names are used to designate the size of the major component in each fraction. K'ase I and II denote keratanase I and II digestion products (further details are in Table S2).

[b] Probes 1-9: only the main components (or 'idealised sequences' in the case of CS-related probes) are shown as representative. Other components with different sulfation contents or patterns exist as the GAG chains are complex and heterogeneous. Probes 10-15: mixtures of oligosaccharide with various sulfation contents/positions as indicated. DH and AO, lipid moieties of NGLs prepared by reductive amination and oxime ligation, respectively (further details are in Table S2).

[c] Only 'idealised sequences' are shown. CS-A and CS-C both contain GalNAc(4S) and GalNAc(6S) but with different ratios. For CS-A the ratio of 4S:6S≈3:2, whereas for CS-C 4S:6S≈1:4, in the respective polysaccharides (Chai W. *et al.* J Biol Chem. 227:22438-22446, 2002).
