# Supplementary material for: Sulfated Glycosaminoglycans as Viral Decoy Receptors for Human Adenovirus Type 37
Source: Viruses. 2019 Mar 12;11(3):247. doi: 10.3390/v11030247 (PMC6466042; doi:10.3390/v11030247)
Supplement: Supplementary file 1 [file viruses-11-00247-s001.zip › viruses-459887-final check-supp/viruses-459887-proofreading done-supplementary.docx]

**Table S2. Supplementary** **Glycan Microarray Document Based on MIRAGE Guidelines** (doi:10.3762/mirage.3)

|  | **Description** |
| --- | --- |
| 1. **Sample: Glycan Binding Sample** | |
| Description of Sample | Recombinant His-tagged HAdV-D37 fiber knob  Methods of preparation and purification are in the main text and the reference below  Burmeister, W.P.; Guilligay, D.; Cusack, S.; Wadell, G.; Arnberg, N. Crystal structure of species D adenovirus fiber knobs and their sialic acid binding sites. J Virol 2004, 78, 7727-7736, ([doi:10.1128/JVI.78.14.7727-7736.2004](https://jvi.asm.org/content/78/14/7727)).  Public database IDs:  GenBank: U69132.1 (The genes corresponding to amino acid residues 177 to 365)  Protein sequence database: >1UXE:SEQUENCE  MRGSHHHHHHGAMGSWNPKYDTRTLWTTPDTSPNCTIAQDKDSKLTLVLTKCGSQILAN  VSLIVVAGKYHIINNKTNPKIKSFTIKLLFNKNGVLLDNSNLGKAYWNFRSGNSNVSTAYE  KAIGFMPNLVAYPKPSNSKKYARDIVYGTIYLGGKPDQPAVIKTTFNQETGCEYSITFNFSW  SKTYENVEFETTSFTFSYIAQE  PDB: 1UXA |
| Sample modifications | Not relevant. |
| Assay protocol | Please see method section in the main text. |
| **2.** **Glycan Library** | |
| Glycan description for defined glycans | The sequence information of the fifteen GAG neoglycolipids (NGL) probes included in the microarray are in **Table S1**. GAG oligosaccharides were prepared by partial depolymerization of the polysaccharides listed below.   \| Poly-saccharides \| Origins/sources \| Reagents used for preparing oligosaccharide fractions \| References \| \| --- \| --- \| --- \| --- \| \| HA \| Bovine vitreous humor  (Sigma H7630) \| Hyaluronate lyase from *Streptomyces hyalurolyticus* (EC 4.2.2.1; Sigma) \| [Chai et al. *Infect Immun.* 2001](https://www.ncbi.nlm.nih.gov/pubmed/11119533) \| \| CSA \| Bovine trachea  Sigma C8529) \| Chondroitin lyase ABC from Proteus vulgaris (EC 4.2.2.4; Sigma) \| [Chai et al. *J Biol Chem.* 2002](https://www.ncbi.nlm.nih.gov/pubmed/11956186) \| \| CSB \| Bovine mucosa  (Sigma C3788) \| \| CSC \| Shark cartilage  (Sigma C4384) \| \| Heparin \| Porcine intestinal mucosa (Sigma H3149) \| Heparin lyase I (EC 4.2.2.7; Sigma) \| [Chai et al. *Anal Chem.* 1998](https://pubs.acs.org/doi/10.1021/ac9712761) \| \| HS \| Porcine intestinal mucosa, Fraction I  (Celsus Laboratories HO-10595) \| Heparin Lyase III (E.C. 4.2.2.8; IBEX Technologies) \| [Chai et al. *Biochemistry,* 2004](https://pubs.acs.org/doi/pdf/10.1021/bi036250k) \| \| KS \| Bovine cornea,  (Gift from Robert Linhardt; Weyers et al., *FEBS J.* [2013](https://www.ncbi.nlm.nih.gov/pubmed/23402351)) \| Keratanase I from *Pseudomonas sp*. (EC 3.2.1.103; Seikagaku);  Keratanase II from *Bacillus circulans.* (EC 3.2.1; [Wang et al., *Glycoconjugate J*. 2017](https://www.ncbi.nlm.nih.gov/pubmed/28752383)) \| [Material intended for publication: 31. Nian Wu, Lisete M. Silva, Yan Liu, Yibing Zhang, Chao Gao, Fuming Zhang, Li Fu, Yanfei Peng, Robert J. Linhardt, Toshisuke Kawasaki, Barbara Mulloy, Wengang Chai, Ten Feizi. Glycan Markers of Human Stem Cells Assigned with Beam Search Arrays. Molecular & Cellular Proteomics, Submitted] \|   The GAG oligosaccharide mixtures were size‐fractionated by gel filtration (Bio-Gel P-6) chromatography, and analyzed by negative‐ion electrospray ionization mass spectrometry after conversion into their ammonium salts for determination of the chain lengths of the major components in the fractions ([Chai et al. *Anal Chem* 1998](https://pubs.acs.org/doi/10.1021/ac9712761)). |
| Glycan description for undefined glycans | Not relevant. |
| Glycan modifications | The GAG NGL probes were prepared from reducing oligosaccharides either by reductive amination with the amino lipid, 1,2-dihexadecyl-sn-glycero-3-phosphoethanolamine [(DHPE) [(Chai et al., *Methods Enzymol.* 2003)](https://www.ncbi.nlm.nih.gov/pubmed/12968363)] or by oxime ligation with aminooxy functionalized DHPE [(AOPE) [(Liu et al*., Chem Biol*. 2007](https://www.ncbi.nlm.nih.gov/pubmed/17656321))]. |
| 1. **3.** **Printing Surface; e.g., Microarray Slide** | |
| Description of surface | Nitrocellulose-coated glass microarray slides. |
| Manufacturer | 16-pad UniSart® 3D Microarray Slide from Sartorius (Gottingen, Germany) |
| Custom preparation of surface | Not relevant. |
| Non-covalent Immobilization | NGLs and glycolipids were formulated as liposomes by adding carrier lipids, phosphatidylcholine (or 1,2-dihexanoyl-*sn*-glycero-3-phosphocholine, designated as DHPC) and cholesterol ([Liu et al., *Methods Mol. Biol.* 2012](https://www.ncbi.nlm.nih.gov/pubmed/22057521)) for robotically arraying and non-covalent immobilization on nitrocellulose-coated glass slides.  Binding signals were glycan dose related. |
| **4. Arrayer (Printer)** | |
| Description of Arrayer | Nano-Plotter 2.1 (GeSim, Radeberg, Germany) |
| Dispensing mechanism | Non-contact liquid delivery with four dispensing tips. |
| Glycan deposition | Approximately 0.33 nL was printed per spot |
| Printing conditions | The printing solutions for NGLs were aqueous-based and contained 100 pmol/μl each of cholesterol and phosphatidylcholine (or DHPC) as lipid carriers in addition to the NGLs, the concentration of which were 5 and 15 pmol/μl for the 2 and 5 fmol per spot levels, respectively.  All printing solutions contained Cy3 NHS ester (GE Healthcare) at 20 ng/ml (26 fmol/μl) as a marker to monitor the printing process.  Printing was performed at ambient temperature and relative humidity of 50-58%. |
| 1. **5.** **Glycan Microarray with “Map”** | |
| Array layout | Each array slide contained 16 identical subarrays (pads). Each subarray contained up to 64 lipid-linked glycans printed at the two levels in duplicate (four spots per saccharide in a row); up to 256 spots (16x16) in total for 64 probes. For the GAG oligosaccharide arrays 15 probes were printed in each subarray. |
| Glycan identification and quality control | The 15 glycan probes printed are defined in Table S1. For quality control, the focused GAG arrays were analysed with various GAG-binding proteins and antibodies, as well as number of viruses and virial proteins. An example using an array contained similar GAG NGL probes is the binding observed with human papillomavirus type 16 ([Cerqueira, et al. *Cell Microbiol.* 2013](https://www.ncbi.nlm.nih.gov/pubmed/23601855)). |
| 1. **6. Detector and Data Processing** | |
| Scanning hardware | GenePix 4300A (Molecular Devices, Berkshire, UK) |
| Scanner settings | Scanning resolution: 10 μm / pixel (this resolution is adequate for the sizes of sample spots)  Laser channel: Red (scan wavelength 635 nm)  PMT voltage: 350  Scan power: Adjusted for each sample to achieve maximum signal without saturation of any single spot. |
| Image analysis software | ScanArray Express software (PerkinElmer LAS, Beaconsfield, UK) for the CLL array and GenePix® Pro 7 (Molecular Devices, Berkshire, UK) for the polysaccharide and KS-related arrays. |
| Data processing | The gpr files were entered into an in-house microarray database using software (designed by Dr Mark Stoll, <http://www.beilstein-institut.de/en/publications/proceedings/glyco-2009>) for data processing. No particular normalization method or statistical analysis was used. |
| **7.** **Glycan Microarray Data Presentation** | |
| Data presentation | The microarray binding results are in Figure 1. Binding results are presented as histogram chart of fluorescence intensity. |
| 1. **8.** **Interpretation and** **Conclusion from Microarray Data** | |
| Data interpretation | No software or algorithms were used to interpret processed data. |
| Conclusions | His-tagged HAdV-D37 fiber knob bound to various sulphated GAG NGLs but not the non-sulfated NGL HA 12-mer. |
